# Logo2PWM: a tool to convert sequence logo to position weight matrix

**DOI:** 10.1186/s12864-017-4023-9

**Published:** 2017-10-03

**Authors:** Zhen Gao, Lu Liu, Jianhua Ruan

**Affiliations:** Department of Computer Science, The University of Texas at San Antonio, One UTSA Circle, San Antonio, 78249 TX USA

**Keywords:** Sequence logo, Position weight matrix, Convert, Motif finding, Transcription, Binding site

## Abstract

**Background:**

position weight matrix (PWM) and sequence logo are the most widely used representations of transcription factor binding site (TFBS) in biological sequences. Sequence logo - a graphical representation of PWM, has been widely used in scientific publications and reports, due to its easiness of human perception, rich information, and simple format. Different from sequence logo, PWM works great as a precise and compact digitalized form, which can be easily used by a variety of motif analysis software. There are a few available tools to generate sequence logos from PWM; however, no tool does the reverse. Such tool to convert sequence logo back to PWM is needed to scan a TFBS represented in logo format in a publication where the PWM is not provided or hard to be acquired. A major difficulty in developing such tool to convert sequence logo to PWM is to deal with the diversity of sequence logo images.

**Results:**

We propose logo2PWM for reconstructing PWM from a large variety of sequence logo images. Evaluation results on over one thousand logos from three sources of different logo format show that the correlation between the reconstructed PWMs and the original PWMs are constantly high, where median correlation is greater than 0.97.

**Conclusion:**

Because of the high recognition accuracy, the easiness of usage, and, the availability of both web-based service and stand-alone application, we believe that logo2PWM can readily benefit the study of transcription by filling the gap between sequence logo and PWM.

## Background

Position weight matrix (PWM), introduced by Stormo et al. [1], is widely used for representing transcription factor binding site (TFBS) in biological sequences. PWMs are often computed from a list of aligned sequences which are potentially functionally related, and have replaced consensus sequences to be the most commonly used TFBS representation in motif discovery software and biological publications. Presented by Schneider et al. [2], sequence logo is a successful graphical representation of PWM/sequence pattern. From a sequence logo, people can easily perceive the information content and the relative frequency of nucleotide for each position of the consensus sequence, therefore can distinguish subtle sequence patterns and significant residues [2, 3]. While sequence logo is good for human perception and understanding, PWM still has advantages over sequence logo in computational field, such as its preciseness and compactness in computer storage; especially PWM is used as the standard format for motif finding and scanning [4].

There are a few tools available for generating sequence logos from PWM or aligned sequences [5–7]; however, currently we have no tool to convert sequence logo back to PWM. In biology publications, the corresponding PWM of a sequence logo may not be found easily. Such tool is especially needed to scan a TFBS represented in sequence logo format in an ‘ancient’ publication where the original PWM is very hard to be acquired. Even if the PWMs are provided by a publication, to have a tool to convert logo to PWM could save time and speedup the motif finding workflow.

In this work, we propose logo2PWM to reconstruct PWMs from sequence logo images, and overcome the major difficulty of reconstructing PWMs from large variety of sequence logo images. Evaluation results on over one thousand logos from three sources with different logo format show that the correlation between the reconstructed PWMs and the original PWMs are constantly high, further support that logo2PWM can be readily used to benefit the study of transcriptional regulatory network.

## Method

### Sequence logo and PWM

**Fig. 1:**
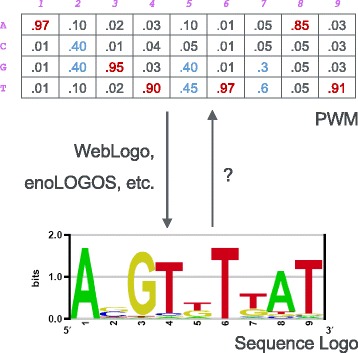
Relationship between sequence logo and PWM

PWM for DNA sequences has four rows, each representing a nucleotide, multiple columns, each representing a TFBS position. Figure 1 shows an example of PWM and its corresponding sequence logo. Here we denote the PWM as *P*. Each element *P*_*ij*_ represents the probability of nucleotide *i*∈{*A,C,G,T*} in position *j*, where the sum probability of each column is 1. To convert a PWM to sequence logo, for each letter column *j*, the height of the whole letter stack is determined by the information content *I* of the column, which is calculated by

$$\begin{array}{*{20}l} I_{j} &= H_{j, max}\left(P_{1}, P_{2}, P_{3}, P_{4}\right) - H_{j}\left(P_{1j}, P_{2j}, P_{3j}, P_{4j}\right) \\&= 2 + \sum_{i=1}^{4}P_{ij}\cdot log_{2}{P_{ij}} \end{array} $$

where *H*_*j*_ represent the entropy of position *j*, and 1 to 4 represents nucleotide A, C, G and T [3, 8]. Then, the height for each nucleotide is calculated by *P*_*ij*_·*I*_*j*_. For the color denotation, usually green, blue, yellow and red represent A, C, G and T respectively.

Reconstruction of the probabilities for nucleotides A, C, G and T from the sequence logo is not as straight forward as it looks. Here we only focus on logo image of one logo-column *j*.

First of all, directly measuring the proportion of each letter of the whole letter-stack at position *j* does not work - the lower the letter stack, the lower the resolution, and therefore the harder to measure the proportion of the letter height. For the cases that one big letter dominates a position, such as logo-columns on position 1, 3, 4, 6 and 9 in Fig. 1, a few pixel difference of the bottom letters will severely influence the accuracy. This phenomenon is worse for the cases that have two similar sized letters and have lower information content, such as column 2, 5, 7, where directly measuring the letter height for probability would cause the reconstructed PWM to have much higher information content. This influence is even worse when the resolution of image is low.

Thus, we utilize the formula of information content to calculate the probabilities. For the cases that only one strong letter is present (the probability of this strong letter is denoted as *p*_1*st*_), we assume that all the three weak letters have equal probability - *p*_*weak*_=(1−*p*_1*st*_)/3. *p*_1*st*_ is estimated based on the information content (height of logo). In this case, information content:

$$I = 2 + p_{1st}\cdot log_{2}p_{1st} + 3 \cdot p_{weak}\cdot log_{2}p_{weak} $$

In order to speed up the slow computational time of solving the above formula, we pre-calculated a *I* to *p*_1*st*_ lookup table, *B*, with *p*_1*st*_ interval 0.01 from 0 to 1.

For the cases that there are two strong letters in the letter-column (such as logo-columns on position 2, 5, 7 in Fig. 1), we consider the height of both the top and the secondary letter, *h*_1*st*_ and *h*_2*nd*_, thus *p*_2*nd*_=*p*_1*st*_∗*h*_2*nd*_/*h*_1*st*_. Here we consider a letter with at least 3 pixel-lines as a strong secondary letter, and we assume that the other two letters have the same probabilities: *p*_*weak*_=(1−*p*_1*st*_−*p*_2*nd*_)/2. The probabilities can be obtained by solving the following equation:

$$I = 2 + p_{1st}\cdot log_{2}{ p_{1st}} + p_{2nd}\cdot log_{2}{ p_{2nd}} + 2 \cdot p_{weak} \cdot log_{2}{p_{weak}} $$

Concatenating the estimated probabilities for all positions, we obtained the reconstructed PWM.

### Algorithm

**Fig. 2:**
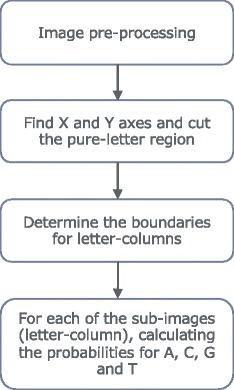
Workflow of core function

Figure 2 shows the workflow of the mian algorithm. Firstly,The image pre-processing module converts the image file to a common three-channel RGB formatted file. Current supported file format includes ‘png’, ‘jpg’, ‘jpeg’ and ‘gif’, except ‘png’ file with alpha channel (transparent channel).

Secondly, the algorithm determines if the logo contains X and Y coordinates using the black pixel feature. We assume that the coordinates in the image are black. If the logo has X and Y coordinates, the algorithm determines the full length and height of the logo, then cuts the pure logo area. If the logo image does not contain X and Y coordinates, the algorithm uses the logo boundary to cut the pure logo area and assume that the highest letter has maximum information content - 2 bits. If the logo image contains Y coordinate, then the height of Y coordinate is used to estimate the information content later. Then the program cuts the pure logo region and removes noise in the image, such as horizontal dashed lines of background patterns in some logo images.

Thirdly, based on the pure logo area, the algorithm determines the sub-image of each letter-column by several image processing algorithms, including:

- *a) Sum the matrix to a 1-D array on X, then count the number of peaks,*
- *b) Vote for consensus gap distances and determine the letter-column width,*
- *c) Use the letter width to determine the number of letters-columns in the logo, then fine-adjust the width of letter.*

Lastly, for each letter-column image, the algorithm estimates the probabilities for letter A, C, G and T. There are two important sub-tasks: letter recognition and probability estimation. To determine the main letter(s) in the sub-image, we used a ‘nearest neighbor’-like algorithm to guess the letter from color. We use the most common color code that green is for A, blue for C, yellow for G and red for T. Six color central points are pre-determined to boost up speed:

$$\begin{array}{llllllr}\textsf{black}: &&{\phantom{00}} \textsf{(0}, &&{\phantom{00}} \textsf{0}, &&{\phantom{00}} \textsf{0)}\\ \textsf{white}: &&{\phantom{00}} \textsf{(255}, &&{\phantom{00}} \textsf{255}, &&{\phantom{00}} \textsf{255)}\\ \textsf{red}: &&{\phantom{00}} \textsf{(200}, &&{\phantom{00}} \textsf{25}, &&{\phantom{00}} \textsf{32)}\\ \textsf{green}: &&{\phantom{00}} \textsf{(57}, &&{\phantom{00}} \textsf{178}, &&{\phantom{00}} \textsf{65)}\\ \textsf{blue}: &&{\phantom{00}} \textsf{(43}, &&{\phantom{00}} \textsf{60}, &&{\phantom{00}} \textsf{147)}\\ \textsf{yellow}: &&{\phantom{00}} \textsf{(240}, &&{\phantom{00}} \textsf{173}, &&{\phantom{00}} \textsf{10)}\\ \end{array} $$

Then for each pixel in the image, the algorithm determines its color by finding its nearest color central point. White pixel is for background, black pixel is for axis and labels/marks, the other four color pixels are for the four nucleotides in DNA sequences. We use consensus voting to determine the color of the letter sub-image. We do not choose to use ‘optical character reader (OCR)’ for the letter recognition task because OCR has low recognition speed and low accuracy for this case, especially due to the uncommon shape of font and the variety of colors.

### Implementation

**Stand-alone application** The core functions of logo2PWM is written in MATLAB 8.6 with image processing toolbox. The complete MATLAB program requires as input the file name of the sequence logo image, and outputs three files in the same folder of the original logo image file: the reconstructed PWM in.csv format, the reconstructed PWM in enologo format, and the Position-specific Scoring Matrix (PSSM) file for MEME suite. The program can also provide the flexibility to accept an optional parameter - the ‘number of columns’, therefore has a higher chance to return a good result.

The source code of stand-alone application can be accessed at http://www.cs.utsa.edu/~jruan/logo2pwm_sa.

**Fig. 3:**
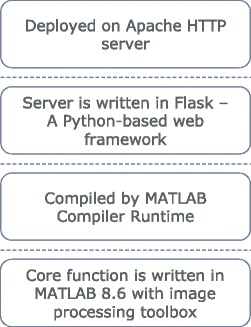
Software architecture of logo2PWM

**Web-based service** As shown in Fig. 3, the software architecture for the web-based service has four layers. From bottom to top, these layers are MATLAB source code, MATLAB compiler runtime executable, web framework, and deployment.

logo2PWM is available at http://www.cs.utsa.edu/~jruan/logo2pwm.

### Evaluation

We evaluate our algorithm by computing the correlation between estimated and true PWMs, and visually examining the original sequence logos and sequence logos regenerated with the estimated PWMs. Three systematic evaluations have been performed on 1946 TFBS logos from - Zhu et al. [9], MacIsaac et al. [10], and, JASPAR-2016 database [11, 12]. There are 179 sequence logo-PWM pairs from Zhu et al. [9], 124 sequence logo-PWM pairs from MacIsaac et al. [10], and 1643 available logo-PWM pairs from the JASPAR-2016 database respectively.

## Results and Discussion

**Fig. 4:**
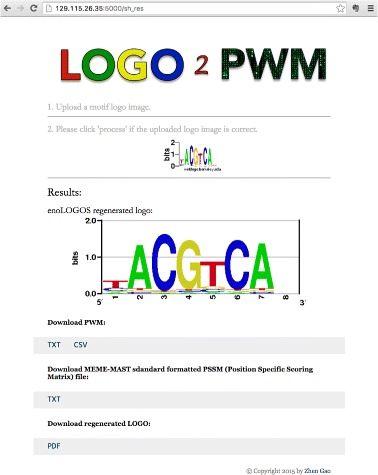
Screen-shot of web-service

Figure 4 shows the index page of the web service. In addition to the stand-alone application, the web tool also automatically re-generates the sequence logo for the estimated PWM using enologos [5].

Currently, execution time for a logo image under MATLAB environment is around 2 s, while the execution time on the web-server is around 15 seconds.

logo2PWM has been tested on a large variety of sequence logo images to validate and improve our algorithm. We also performed three systematic evaluations on over one thousand TFBS logos from - Zhu et al. [9], MacIsaac et al. [10], and the JASPAR-2016 database [11] - results for all three data sets are good.

### Evaluation on 179 logos from Zhu et al. [9]

**Fig. 5:**
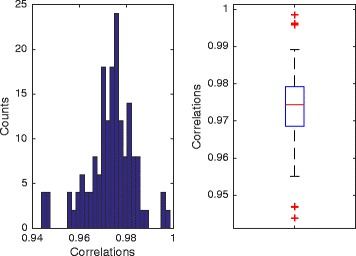
Evaluation on 179 logos from Zhu et al. [9]

**Fig. 6:**
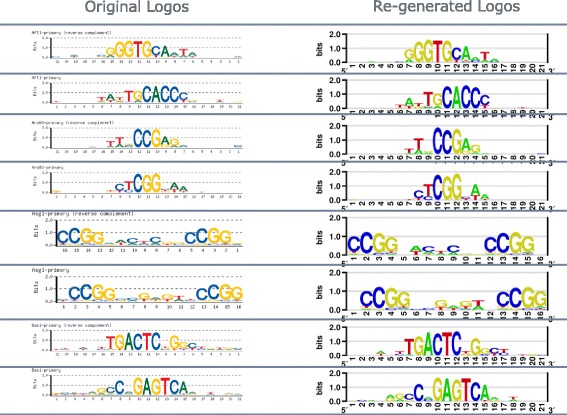
Visual comparison between re-generated and original logos on arbitrarily selected logos from Zhu et al. [9]

On 179 logos from Zhu et al. [9], our estimated PWMs has high correlation of the original PWMs all-across the sequence logo images (Fig. 5). For all logos, the estimated PWM correlation is greater than 0.94, and the median is above 0.97. Visual comparison between re-generated and original logos on arbitrarily selected logos is almost identical, shown in Fig. 6.

### Evaluation on 124 logos from MacIsaac et al. [10]

**Fig. 7:**
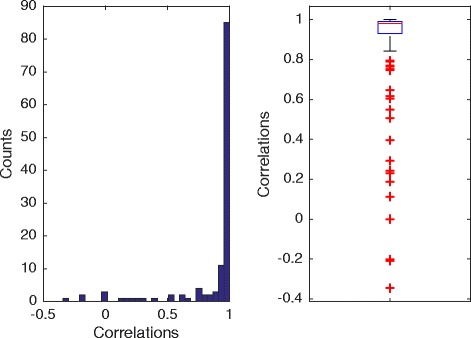
Evaluation on 124 logos from MacIsaac et al. [10]

**Fig. 8:**
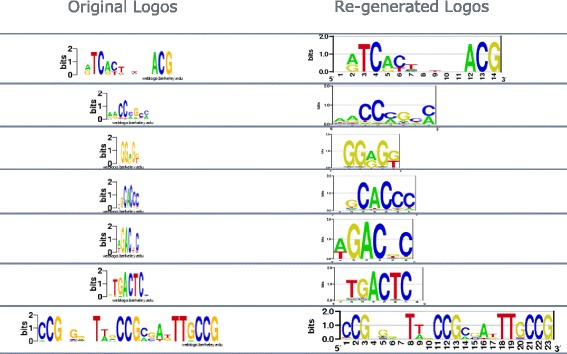
Visual comparison between re-generated and original logos on arbitrarily selected logos from MacIsaac et al. [10]

Figure 7 shows the distribution of correlation between 124 estimated PWMs and their original PWMs. Our algorithm works well on most of logos. The median correlation is 0.98. The outliers shown by the red ‘+’ signs on the boxplot indicates the failed cases. Actually, most of the failed cases are due to bad logo images - such logo images cannot even read by human. For example, sequence logos with letter width less than one pixel (multiple cases in this dataset). Visual comparison between re-generated and original logos on arbitrarily selected logos shows no obvious flaw (Fig. 8).

### Evaluation on 1,643 logos from the JASPAR-2016 database

**Fig. 9:**
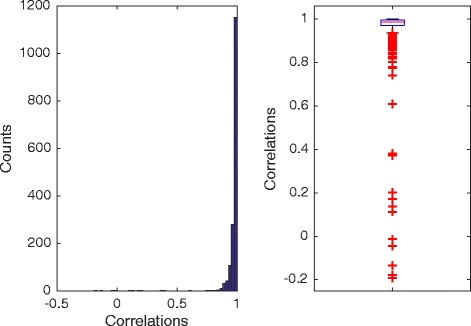
Evaluation on 1643 logos from the JASPAR-2016 database

**Fig. 10:**
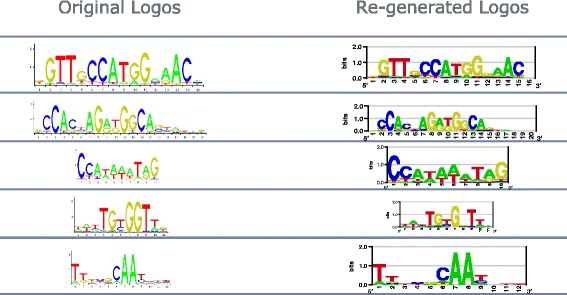
Visual comparison between re-generated and original logos on arbitrarily selected logos from the JASPAR-2016 database

The JASPAR-2016 database provides entries for 2049 TFs of variety kinds of species. Within the 2049 TF-entries, 1643 of them have their sequence logo available. Figure 9 shows the distribution of correlation between the 1643 estimated PWMs and their original PWMs. Our algorithm still works fluently on most of logos. The median correlation is 0.99. Visual comparison on Fig. 10 shows that our logo2PWM tool also handles the JASPAR logo format well.

**Fig. 11:**
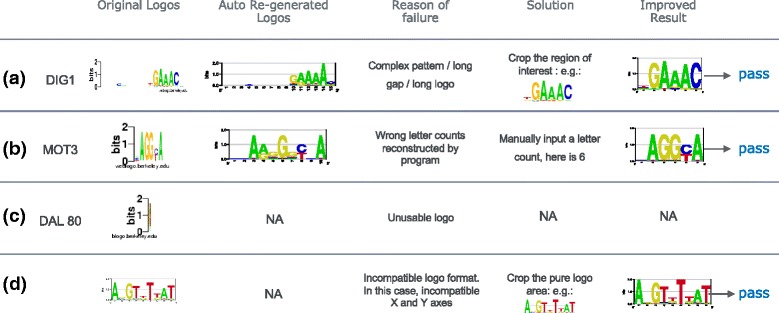
Failure cases and solutions. (**a**) to (**d**) show four types of failure cases and the corresponding solutions

To accurately estimated PWMs from a large variety of sequence logo images is a challenging task, and sometimes the automatic option of our tool fails. Usually, passing the number of letter-columns parameter *n* and carefully cropping the pure logo region of the logo image solve most of problems. Figure 11 shows four examples of failures using the default options of logo2PWM. Figure 11 a shows a good example of a sequence logo with a long gap, which make the pattern complex and the PWM hard to be reconstructed. Our solution is to crop the region of interest. Note that logo2PWM works best with no left and right margin on the logo image. Sometimes, the resolution of the image is so low (Fig. 11 b) that the number of letters cannot be correctly counted by program. In this case, we simply need to input a letter count parameter *n* to the tool. There are also several cases just like Fig. 11 c, where bad logos cause the failure of the tool, which lower down the overall evaluation accuracy shown in Fig. 7. Figure 11 d shows an example of logo2PWM’s failure dues to incompatible logo format, which can be easily solved by cropping logo image.

## Conclusions

We proposed logo2PWM to reconstruct PWM from sequence logo images. Based on the decent evaluation results on over one thousand logos images from variety of logo format, the easiness of usage, and, the availability of both web-based service and stand-alone application, we believe that logo2PWM can readily benefit the study of TF-DNA interaction.
